# Adsorption of Tea Polyphenols using Microporous Starch: A Study of Kinetics, Equilibrium and Thermodynamics

**DOI:** 10.3390/molecules24081449

**Published:** 2019-04-12

**Authors:** Xianchun Hu, Xianfeng Du

**Affiliations:** 1 State Key Laboratory of Tea Plant Biology and Utilization, Anhui Agricultural University, Hefei 230036, China; tea30@163.com; 2 College of Horticulture and Gardening, Yangtze University, Jingzhou 434025, China

**Keywords:** microporous starch, adsorption, tea polyphenols, kinetics, isotherm, thermodynamics

## Abstract

Microporous starch (MPS) granules were formed by the partial hydrolysis of starch using α–amylase and glucoamylase. Due to its biodegradability and safety, MPS was employed to adsorb tea polyphenols (TPS) based on their microporous characteristics. The influences of solution pH, time, initial concentration and temperature on the adsorptive capacity were investigated. The adsorption kinetics data conformed to the pseudo second–order kinetics model, and the equilibrium adsorption data were well described by the Langmuir isotherm model. According to the fitting of the adsorption isotherm formula, the maximum adsorption capacity of TPS onto MPS at pH 6.7 and T = 293 K was approximately 63.1 mg/g. The thermodynamic parameters suggested that the adsorption of TPS onto MPS was spontaneous and exothermic. Fourier transform infrared (FT–IR) analysis and the thermodynamics data were consistent with a physical adsorption mechanism. In addition, MPS-loaded TPS had better stability during long-term storage at ambient temperature.

## 1. Introduction

Tea polyphenols (TPS) are one of the main active substances in tea leaves [1,2]. Tea polyphenols possess aromatic rings with one or more hydroxyl groups [3], which have been shown to provide significant health benefits, including antioxidant activity, anticancer activity, reducing blood fat, and lowering blood sugar; hence, TPS are widely applicable in cosmetics, clinical applications, drugs, and food [4,5,6,7].

Microporous starch (MPS) is prepared by reacting various raw starches with starch hydrolytic enzymes (α–amylase and glucoamylase) at temperatures under the gelatinization point [8,9,10,11,12,13]. Microporous starch granules have plentiful micro pores expanding inside (hilum) from outside (surface), which allow smaller molecules to enter the holes of the granules [14]. Therefore, microporous starch has remarkable adsorption performance because of its large specific surface area. It can be used as an adsorbent in food, medicine, cosmetics, agriculture and other fields [15,16,17,18]. For example, some easily oxidized substances (such as vitamin E, β–carotene, lycopene, etc.) can be protected efficiently by the adsorption of microporous starch [18].

Tea polyphenols are susceptible to oxidants, light, and heat. Microporous starches having abundant pores that can absorb and protect the TPS. To the best of our knowledge, the effects of the kinetics, isotherm behavior and thermodynamics on the adsorption of tea polyphenols in/on MPS have not been investigated. The evaluation of these parameters would be useful for comprehending the types of adsorption occurring and the adsorption mechanisms [19]. The adsorption kinetics and adsorption isotherms are the essential aspects to monitor during the process of adsorption [20]. The thermodynamic parameters, including enthalpy change, entropy change and free energy change, provide profound information on the intrinsic energetic changes that are connected with adsorption [21,22,23].

The objectives of this study were to prepare a microporous starch adsorbent by modification and to assess the kinetics, isotherms and thermodynamics of TPS adsorption onto MPS. The adsorption rates are assessed with the pseudo first–order, pseudo second–order and Weber and Morris intra–particle diffusion equations. The effect of temperature on the adsorption isotherms is measured, and the thermodynamic parameters of free energy (∆G^0^), enthalpy (∆H^0^) and entropy (∆S^0^) during adsorption at different temperatures are calculated. Kinetic and equilibrium isotherm models were employed to evaluate the rate of adsorption and adsorption capacity as well as to reveal the mechanism of TPS adsorption.

## 2. Results and Discussion

### 2.1. Effect of pH

**Figure 1:**
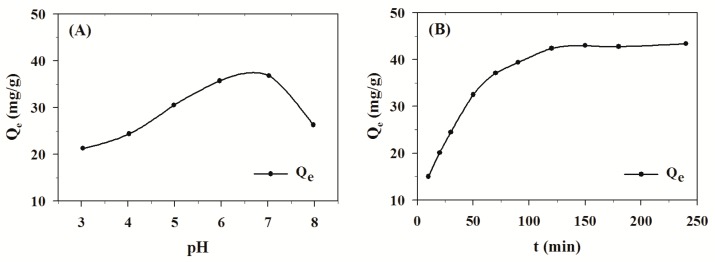
Effect of pH (**A**) and time (**B**) on the adsorption of tea polyphenols (TPS) by microporous starch (MPS) (MPS dose = 1.0 g/50 mL, Temp. = 293 K).

The adsorption behavior of TPS onto MPS may differ due to pH values. For this reason, experiments were conducted in different solutions ranging from pH 3 to pH 8, see Figure 1A (Initial conc. = 1.0 mg/mL, Adsorption time = 150 min). The pH of the mixture solution is an important limiting factor in the TPS adsorption process and, consequently, it is necessary to optimize the pH of the solution to obtain effective interactions between TPS and MPS. Previous studies confirmed that pH value had great influence on the interaction between different polyphenols and macromolecule materials [24,25]. In this context, a higher adsorption capacity of TPS onto MPS was obtained by optimizing the pH of the adsorption solution. As shown in Figure 1A, the Q_e_ of TPS increased with pH up to 6.7 and then began to decrease. The maximum adsorption of TPS onto MPS was obtained at pH 6.7; thus, this pH value was used for the following research. 

### 2.2. Effect of Contact Time

The static adsorption curve was studied to obtain the most suitable adsorption time. The result is presented in Figure 1B (Initial conc. = 1.2 mg/mL, pH = 6.7). The adsorption capacity quickly increased to 39.4 mg/g within 90 min at 293 K. With the increase in contact time, adsorption equilibrium was achieved at approximately 120 min and reached a plateau at an adsorption capacity of 42.4 mg/g. Therefore, the most appropriate adsorption time was 120 min. The result obtained from this experiment was further used to fit the adsorption kinetic models.

### 2.3. Adsorption Kinetics

Adsorption kinetics describe the adsorption rate of the adsorbate on an adsorbent at a specific initial concentration and temperature, and the time required for the adsorption from the beginning of the reaction to equilibrium is determined from the kinetics [26]. For a solid–liquid adsorption process, the solute transfer is usually characterized by either external mass transfer or intra–particle diffusion or both [27]. Various kinetics models have been proposed for adsorption, such as the pseudo first–order, the pseudo second–order and Weber and Morris intra–particle diffusion kinetics models, etc.

#### 2.3.1. Pseudo First-Order Model

The Lagergren rate equation is one of the most widely used adsorption rate equations to describe adsorption processes. The pseudo first–order equation can be expressed in the following form, given as Equation (1) [28]:

(1) $$\frac{dQ_{t}}{dt}=k_{f}(Q_{e}-Q_{t})$$

Equation (1) can be integrated as follows:

(2) $$\log(Q_{e}-Q_{t})=\log Q_{e}-\frac{k_{f}}{2.303}t$$

where t is the contact time (min), and Q_t_ (mg/g) and Q_e_ (mg/g) are the quantities of TPS absorbed at time t and at equilibrium, respectively. k_f_ is the rate constant of the pseudo first–order model (1/min). 

**Figure 2:**
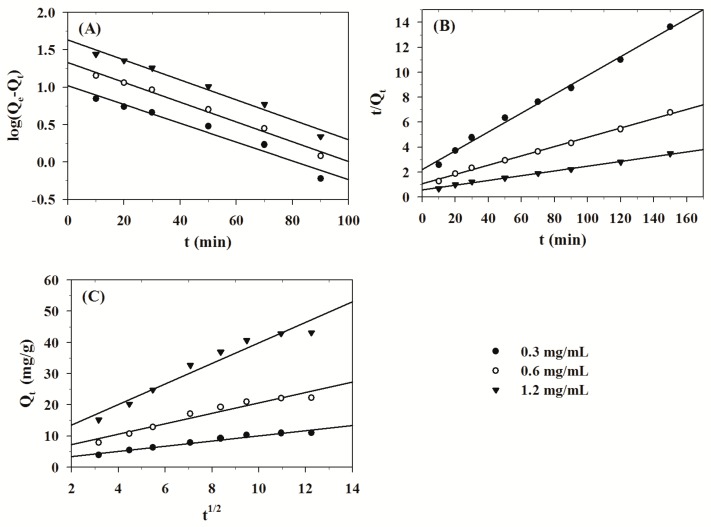
Pseudo first–order kinetic plot (**A**), pseudo second–order kinetic plot (**B**) and Weber and Morris intra–particle diffusion kinetic model (**C**) for the adsorption of TPS onto MPS (MPS dose = 1.0 g/50 mL, pH = 6.7, Initial conc. = 0.3–1.2 mg/mL, Temp. = 293 K).

Plotting log (Q_e_ − Q_t_) versus t allows the calculation of the rate constant k_f_ and Q_e_. The result is shown in Figure 2A. The linear plot displays the effectiveness of this model [28]. The linear correlation coefficients (R^2^) of the initial concentration of 0.3 mg/mL, 0.6 mg/mL and 1.2 mg/mL were 0.96015, 0.98879 and 0.97810, respectively. Kinetics parameter k_f_ suggested that the adsorption rate was very fast at the beginning of the adsorption process for TPS onto MPS.

#### 2.3.2. Pseudo Second-Order Model

The pseudo second–order equation assumed that the adsorption rate was proportional to the number of active sites occupied onto the adsorbent [29]. The pseudo-second-order model is given by Equation (3) [30]:

(3) $$\frac{dQ_{t}}{dt}=k_{s}{\left(Q_{e}-Q_{t}\right)}^{2}$$

Equation (3) can be rearranged as follows:

(4) $$\frac{t}{Q_{t}}=\frac{1}{k_{s}Q_{e}^{2}}+\frac{1}{Q_{e}}t$$

where t is the contact time (min), and Q_t_ (mg/g) and Q_e_ (mg/g) are the quantities of TPS absorbed at time t and at equilibrium, respectively. k_s_ is the rate constant of the pseudo second-order model (g/mg min).

Q_e_ and k_s_ can be calculated from a plot of t/Q_t_ versus t. The result is shown in Figure 2B. The linear correlation coefficients (R^2^) of the initial concentration of 0.3 mg/mL, 0.6 mg/mL and 1.2 mg/mL were 0.99471, 0.99640 and 0.99586, respectively. The straight linearity suggested that the pseudo second-­order model was fit for describing the adsorption kinetics of TPS onto MPS. The correlation coefficient of the pseudo second-order model was higher than the pseudo first-order model, which may imply that the pseudo second-order is more suitable to describe the kinetics.

#### 2.3.3. Weber and Morris Intra-Particle Diffusion Model

Adsorption kinetics were controlled by the adsorption mechanism and rate-limiting step of the process [31]. The adsorption rate can be limited by film diffusion and/or intra-particle diffusion [32]. The intra-particle diffusion model can be expressed as follow:

(5) $$Q_{t}=k_{id}t^{1/2}+C$$

where Q_t_ (mg/g) is the quantities of TPS absorbed at time t, and k_id_ is the intra–particle diffusion rate constant. The values of the intercept, C, provide an indication of the thickness of the boundary layer between the adsorbent and adsorbate. The larger the value of C, the greater the boundary layer effect is.

The linear correlation coefficients (R^2^) of the initial concentration of 0.3 mg/mL, 0.6 mg/mL and 1.2 mg/mL were 0.96317, 0.94311 and 0.94871, respectively. The result is presented in Figure 2C. If the plot shows a straight line that passes through the origin, the adsorption process is conducted only by intra-particle diffusion. If the plot does not pass through the origin, the adsorption process is conducted by two or more diffusion mechanisms [33,34]. The result showed that the straight lines did not pass through the origin, suggesting that both film diffusion and intra-particle diffusion were significant between MPS and TPS.

### 2.4. Adsorption Isotherm

Adsorption isotherms describe the adsorbate distribution between that adsorbed onto the adsorbent and that in solution when adsorption equilibrium is achieved at a steady temperature [35,36]. At present, the most common models are the Langmuir, Freundlich and Tempkin isotherms. Consequently, the Langmuir, Freundlich and Temkin isotherm models were employed to describe the equilibrium characteristics of adsorption of TPS onto MPS. 

#### 2.4.1. Langmuir Isotherm

**Figure 3:**
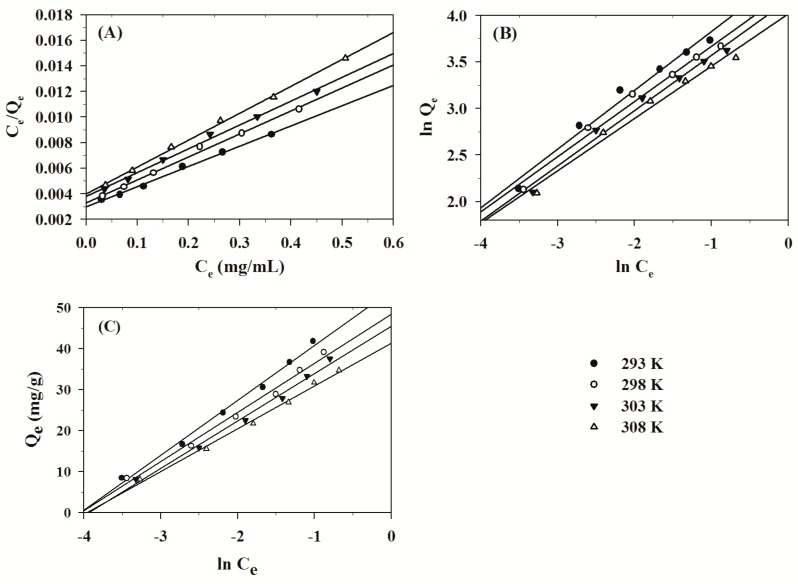
Langmuir adsorption isotherm (**A**), Freundlich adsorption isotherm (**B**) and Tempkin adsorption isotherm (**C**) for the adsorption of TPS onto MPS (MPS dose = 1.0 g/50 mL, pH = 6.7, Adsorption time = 120 min, Initial conc. = 0.2–1.2 mg/mL, Temp. = 293–308 K).

The Langmuir isotherm is given for homogenous adsorption where the adsorption process has equal activation energy. The Langmuir isotherm can be represented as follows [37]:

(6) $$\frac{C_{e}}{Q_{e}}=\frac{1}{K_{L}Q_{m}}+\frac{C_{e}}{Q_{m}}$$

where Q_e_ is the equilibrium quantity of absorbed TPS (mg/g), C_e_ is the equilibrium concentration (mg/mL), Q_m_ is the calculated maximum quantities of absorbed TPS, and K_L_ is the Langmuir constant. A plot of C_e_/Q_e_ versus C_e_ is a straight line with intercept 1/Q_m_K_L_ and slope 1/Q_m_. The maximum adsorption capacity of TPS onto MPS at the temperature of 293 K was approximately 63.1 mg/g. Langmuir plot for the adsorption of TPS onto MPS is presented in Figure 3A.

The linear correlation coefficients (R^2^) of the temperature of 293 K, 298 K, 303 K and 308 K were 0.99600, 0.99435, 0.99519 and 0.99820, respectively. The correlation coefficient (R^2^ ≥ 0.99435) of the Langmuir equation was high, which implied that the Langmuir isotherm model was favorable. In addition, the essential characteristics of the Langmuir isotherm can be made on the basis of a dimensionless constant equilibrium, that is separation factor R_L_ [38,39], which is defined by:

(7) $$R_{L}=\frac{1}{1+K_{L}C_{0}}$$

where K_L_ (L/mg) and C_0_ (mg/mL) are the Langmuir constant and the initial concentration of TPS, respectively. The value of R_L_ suggest whether the isotherm is unfavorable (R_L_ > 1), linear (R_L_ = 1), favorable (0 < R_L_ < 1), or irreversible (R_L_ = 0) [39]. The values of R_L_ at 293 K, 298 K, 303 K and 308 K were 0.09579 – 0.01735, 0.07776 – 0.01386, 0.06605 – 0.01165 and 0.05612 – 0.00981, respectively. The values of R_L_ also implied that the Langmuir isotherm was favorable.

#### 2.4.2. Freundlich Isotherm

The Freundlich isotherm model characterizes the adsorption process based on heterogeneous surfaces [31]. The Freundlich isotherm can be represented as follows:

(8) $$\ln Q_{e}=\ln K_{F}+\frac{1}{n}\ln C_{e}$$

where Q_e_ is the equilibrium quantity of absorbed TPS (mg/g), C_e_ is the equilibrium concentration (mg/mL), and K_F_ and 1/n are the Freundlich constants.

The linear plot of ln Q_e_ versus ln C_e_ is shown in Figure 3B. The linear correlation coefficients (R^2^) of the temperature of 293 K, 298 K, 303 K and 308 K were 0.97858, 0.98307, 0.98418 and 0.98190, respectively. The high correlation coefficient (R^2^ ≥ 0.97858) suggested that the Freundlich adsorption isotherm was suitable. The values of 1/n at 293 K, 298 K, 303 K and 308 K were 0.63022, 0.59253, 0.59263 and 0.56199, respectively. The value of 1/n suggests the type of isotherm. When 1 > 1/n > 0, the adsorption isotherm is favorable; when 1/n = 1, the adsorption isotherm is irreversible; and when 1/n > 1, the adsorption isotherm is unfavorable [34,35]. Consequently, the value of 1/n also implied that the adsorption of TPS onto MPS was favorable. 

#### 2.4.3. Tempkin Isotherm

The assumption behind the Tempkin isotherm is that the heat of adsorption of all molecules in the layer decrease with coverage due to adsorbate–adsorbate interaction. The Tempkin isotherm is given by Equation (9) [33,34]:

(9) $$Q_{e}=\frac{RT}{b}\ln(K_{T}C_{e})$$

Equation (9) can be integrated as follows:

Q_e_ = B_1_ ln K_T_ + B_1_ ln C_e_ (10)

where B_1_ = RT/b, K_T_ is the Tempkin isotherm energy constant (L/mg) that corresponds to the maximum binding energy and constant B_1_ is related to the heat of adsorption. Regression of Q_e_ against ln C_e_ enables the calculation of the isotherm constants K_T_ and B_1_. The plot of Q_e_ versus ln C_e_ is shown in Figure 3C. The linear correlation coefficients (R^2^) of the temperature of 293 K, 298 K, 303 K and 308 K were 0.99198, 0.98919, 0.98991 and 0.99359, respectively. The high correlation coefficient (R^2^ ≥ 0.98919) indicated that the Tempkin adsorption isotherm was suitable.

All adsorption isotherms of the Langmuir, Freundlich and Tempkin are shown in Figure 3. From the figure it is clear that all the evaluated equilibrium isotherm models produced good fits for the experimental data. Compared to the Langmuir isotherm model, the average correlation coefficient of the Freundlich and Tempkin isotherm models were lower, and consequently the Langmuir isotherm model may be more suitable for the process of adsorption in this study. Based on the results of the study, the best isotherm models fitted for the adsorption of TPS onto MPS were determined in the following order: Langmuir > Tempkin > Freundlich.

### 2.5. Thermodynamic Study of Adsorption

**Figure 4:**
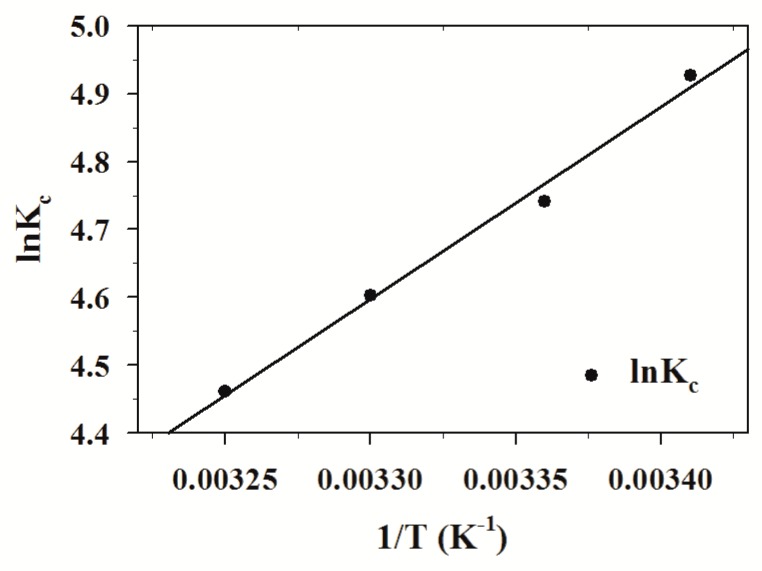
Plots of ln K_c_ versus 1/T for the adsorption of TPS onto MPS (MPS dose = 1.0 g/50 mL, pH = 6.7, Adsorption time = 120 min, Initial conc. = 1.0 mg/mL, Temp. = 293–308 K).

Thermodynamic parameters can describe the thermodynamic behavior associated with TPS adsorption onto MPS from an aqueous solution. Therefore, the change in free energy (∆G^0^), enthalpy (∆H^0^) and entropy (∆S^0^) were determined using the following equations [40]:

(11) $$K_{c}=\frac{Q_{e}}{C_{e}}$$

(12) $$\ln K_{c}=\frac{\Delta H^{0}}{RT}+\frac{\Delta S^{0}}{R}$$

(13) $$\Delta G^{0}=\Delta H^{0}-T\Delta S^{0}$$

where K_c_ is the distribution coefficient (mL/g); Q_e_ and C_e_ are the equilibrium quantities of absorbed TPS (mg/g) and the equilibrium concentration (mg/mL), respectively; and T is the absolute temperature in Kelvin; and R is the ideal gas constant (8.314 J/mol K). The parameters of ∆H^0^ and ∆S^0^ can be calculated from the slope and intercept of the plot of ln K_c_ versus 1/T (Figure 4). The thermodynamic parameters calculated for the adsorption of TPS onto MPS are summarized in Table 1. 

The values of ∆G^0^ tended to be negative as temperature decreases, suggesting that the adsorption process is more suitable at a lower temperature. The negative value of ∆H^0^ and ∆G^0^ confirmed the exothermic and spontaneous nature of the adsorption process [40]. In addition, the values of ∆G^0^ are in the range of −20 to 0 kJ/mol K, indicating that the adsorption of TPS onto MPS is typically via physical adsorption [41]. It was reported that the energy of adsorption from hydrogen bond forces was in the range of 2 to 40 kJ/mol [42]. The value (−23.0526 kJ/mol) of ∆H^0^ suggested that hydrogen bonding interaction between TPS and MPS played an important role in the adsorption process. The negative value of ∆S^0^ implied that the randomness decreased at the solid–solution interface during the adsorption of TPS onto MPS.

### 2.6. The Stability of Microporous Starch (MPS)-Loaded Tea Polyphenols (TPS)

**Figure 5:**
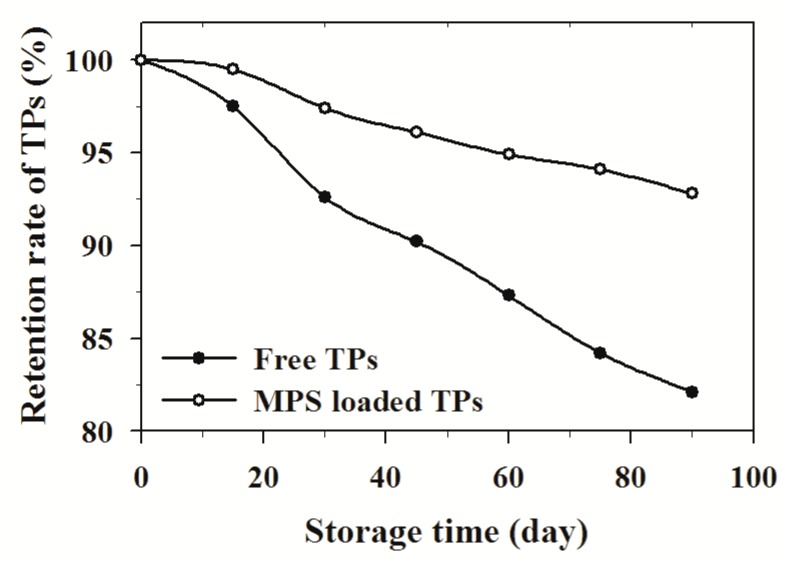
Storage stability of TPS adsorbed onto MPS at ambient temperature.

The samples were stored at ambient temperature, under dry conditions. The stabilities of free TPS and MPS-loaded TPS are shown in Figure 5. From 1 to 15 days, the TPS retention rate value of free TPS changed less. Following an increase in storage time from 15 to 90 days, the retention rate values of free TPS decreased rapidly. The whole reducing ratio from 1 to 90 days relative to the initial quantity was approximately 18.2%, whereas that of MPS-loaded TPS was minor, which was 8.7%. The change in retention rate value of TPS showed that the retention rate of TPS for MPS-loaded TPS was significantly different. Therefore, MPS-loaded TPS had better stability and could be stored for a long time.

### 2.7. Characterization of the Adsorbents

**Figure 6:**
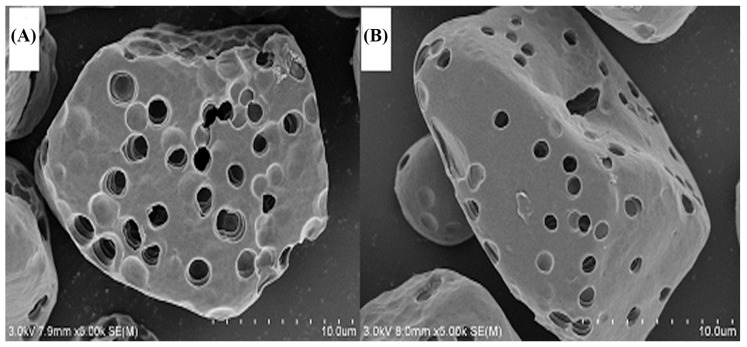
Scanning electron microscope (SEM) images of MPS before (**A**) and after (**B**) adsorption of TPS, magnified 5000 times.

It is well–known that the surface morphology of the adsorbent can affect its adsorption performance [43,44,45]. Figure 6 shows scanning electron microscope (SEM) images obtained for MPS before and after the adsorption of TPS. From the SEM images, the microporous starch granules have polygonal, granular shapes with relatively smooth surfaces. MPS-loaded TPS did not change the surface morphology of the granular starch. However, the pore diameter of starch decreased or nearly disappeared after adsorption, suggesting that TPS molecules diffused toward the pores of MPS and, therefore, were adsorbed. The adsorption performance of MPS mainly depends on microporous characteristics. Therefore, the adsorption capacity of MPS is proportional to specific surface area. Being protected by the crust of the microporous starch, the adsorbed TPS cannot easily oxidize and the microporous starch shows good protection performance.

**Figure 7:**
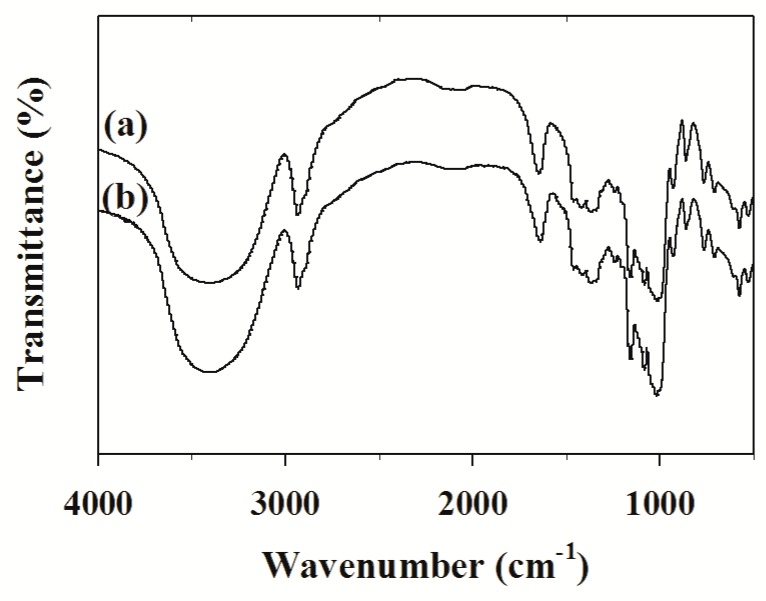
Fourier transform infrared (FT–IR) spectra of MPS before (a) and after (b) adsorption of TPS.

The Fourier transform infrared (FT–IR) spectra of MPS before and after the adsorption of the TPS also indicated these interactions (Figure 7). The characteristic absorption peaks of MPS have no obvious change before and after the adsorption of TPS, which is attributed to the fact that the adsorption process does not result in a change in the molecular structure of the starch [46]. Therefore, the infrared (IR) analysis shows that there are no new chemical bonds formed between TPS and MPS. The results suggested that TPS was adsorbed on MPS mainly through the formation of hydrogen bond complexes with functional groups on the microporous starch surface, which is consistent with the results obtained above.

## 3. Materials and Methods

### 3.1. Materials

Corn starch (food-grade) was obtained from Shandong Linghua Group Co., Ltd., (Jining, China). α–amylase (activity ≥3.7 U/mg) and glucoamylase (activity ≥ 100 U/mg) were purchased from Beijing Aoboxing Biotechnology Co., Ltd., (Beijing, China). Tea polyphenols (TPS, concentration ≥98%) were purchased from Shanghai Winherb Medical Technology Co., Ltd., (Shanghai, China), and were used without further purification. Folin–Ciocalteu reagent was purchased from Sigma-Aldrich Co., Ltd., (Shanghai, China). Disodium hydrogen phosphate, citric acid and ethanol were analytical reagent grade and applied as received. All reagents were prepared using distilled water. 

### 3.2. Preparation of Microporous Starch

Microporous starch (MPS) was prepared by hydrolyzing the native corn starch with α–amylase and glucoamylase [43]. Approximately 25 g native corn starch and 0.5 g admixture of α–amylase and glucoamylase (6:1, *w*/*w*) were suspended in 200 mL phosphate buffer saline (PBS) at pH 5.5 (0.2 M disodium hydrogen phosphate, 0.1 M citric acid) and stirred at 50 °C for 12 h. Then, the mixture was adjusted to pH 10 with 1 mol/L NaOH and was filtered through the suction filter and washed with distilled water four times; the product was dried via vacuum freeze drying and was finally ground and sifted with a vibrating screen (100 meshes) for separation.

### 3.3. Determination of Tea Polyphenols Content

The amount of tea polyphenols was determined by Folin–Ciocalteu’s colorimetric method with slight modification [47,48]. Tea polyphenol aqueous solutions with concentrations ranging from 10 to 60 μg/mL were employed to establish the calibration curve (y = 0.0108x + 0.037; R^2^ = 0.9972). In this method, 1.0 mL of sample was diluted with distilled water to obtain absorbance in the scope of the obtained calibration curve. Thereafter, 5.0 mL 10% Folin–Ciocalteu reagent was added and allowed to stand for 5 min for the reaction. The mixture was regulated by 4.0 mL 7.5% sodium carbonate and was kept in dark place at room temperature (60 min). The absorbance of the resulting blue color was determined at 765 nm using an ultraviolet–visible (UV/VIS) spectrometer (Lambda 35 PerkinElmer, Waltham, MA, USA) (28). The quantitative calculation was carried out according to the calibration curve of TPS.

### 3.4. Batch Adsorption Experiments

Approximately 1.0 g MPS was added to 50 mL aqueous TPS solution in a series of 100 mL glass–stopper Erlenmeyer flasks. The mixture was stirred on a magnetic stirring apparatus at a constant speed of 120 rpm in a thermostat water bath. After a certain adsorption time, the mixture was centrifuged for 10 min at 8,000 rpm. The concentration of TPS in the supernatant solution was measured using a UV/VIS spectrometer (Lambda 35 PerkinElmer, Waltham, MA, USA) at 765 nm.

The adsorption capacity was calculated according to Equation (14):

(14) $$Q=\frac{(C_{i}-C_{t})V}{W}$$

where *Q* is the adsorption capacity of MPS (mg/g), and *C*_i_ and *C*_t_ (mg/L) are the initial and terminal concentrations of TPS in the adsorption solution, respectively. *V* (mL) is the volume of the adsorption solution and *W* (mg) is the mass of the adsorbent.

The pH effect was employed by the method of batching adsorption as mentioned above. The initial concentration of TPS was 1.0 mg/mL. The pH was adjusted from 3 to 8 by regulation of 0.1 mol/L HCl or NaOH. After it was stirred for 150 min, the mixture was subsequently centrifuged for 10 min at 8,000 rpm for the separation of solid phases from the aqueous phase. The concentration of TPS was determined according to 3.3. All experiments were repeated three times.

### 3.5. The Stability Evaluation of MPS-Loaded TPS

The samples were stored at ambient temperature, in dry conditions and were assayed for the retention of TPS every 15 days to study the storage stability of TPS. The desorption of MPS-loaded TPS was performed by adding 50 mL of an aqueous ethanol mixture (70%, *v*/*v*) [49]. The solution was again stirred under the same conditions as in the absorption. Experiments were performed with free TPS as blank controls. The retention rates of the TPS are calculated according to Equation (15):

(15) $$R(\%)=\frac{C_{a}}{C_{b}}\times 100\%$$

where C_b_ and C_a_ are the content of TPS in the sample before treatments and after the treatments, respectively.

### 3.6. Characterization Methods

The morphologies of MPS and MPS-loaded TPS were investigated using a scanning electron microscope (SEM, S-3800N, Hitachi Limited, Tokyo, Japan). FT–IR analyses of MPS and MPS-loaded TPS were performed using Fourier transform infrared spectroscopy (Nicolette is50, Thermo Fisher Scientific, Waltham, MA, USA). The samples were prepared by making pellets with dried IR-grade KBr and 64 scans were signal-averaged to reduce spectral noise.

## 4. Conclusions

Microporous starch (MPS) granules were prepared via the partial hydrolysis of starch using α–amylase and glucoamylase. The results of scanning electron microscopy (SEM) analysis revealed the microporous structure of the MPS. As an adsorbent of tea polyphenols (TPS), MPS shows a much higher adsorption capacity under the right conditions. The adsorption kinetics of TPS onto MPS conforms to the pseudo second-order model. The equilibrium adsorption data were well-fitted by the Langmuir isotherm model. The ∆G^0^ values of the adsorption process become less negative as temperature increased from 293 K to 308 K, and all values of ∆H^0^ are negative, which implies that the adsorption process is exothermic and spontaneous. The FT–IR analysis shows that there are no new chemical bonds formed between TPS and MPS, and the small values of ∆G^0^ between –20 and 0 kJ/mol reveal that the adsorption mechanism of TPS onto MPS is typical physical adsorption. In addition, MPS-loaded TPS had better stability during long-term storage at ambient temperature.

## Figures and Tables

**Table 1:** Thermodynamic parameters for the adsorption of TPS onto MPS.

T (K)	∆G^0^ (kJ/mol K)	∆H^0^ (kJ/mol)	∆S^0^ (J/mol K)
293	−11.9748	−23.0526	−37.8082
298	−11.7858		
303	−11.5967		
308	−11.4077		
